# Are Maternal Antibodies Really That Important? Patterns in the Immunologic Development of Altricial Passerine House Sparrows (*Passer domesticus*)

**DOI:** 10.1371/journal.pone.0009639

**Published:** 2010-03-11

**Authors:** Marisa O. King, Jeb P. Owen, Hubert G. Schwabl

**Affiliations:** 1 School of Biological Sciences, Washington State University, Pullman, Washington, United States of America; 2 Department of Entomology, Washington State University, Pullman, Washington, United States of America; Pennsylvania State University, United States of America

## Abstract

**Background:**

Maternal antibodies are believed to play an integral role in protecting immunologically immature wild-passerines from environmental antigens. This study comprehensively examines the early development of the adaptive immune system in an altricial-developing wild passerine species, the house sparrow (*Passer domestics*), by characterizing the half-life of maternal antibodies in nestling plasma, the onset of *de novo* synthesis of endogenous antibodies by nestlings, and the timing of immunological independence, where nestlings rely entirely on their own antibodies for immunologic protection.

**Methodology/Principal Findings:**

In an aviary study we vaccinated females against a novel antigen that these birds would not otherwise encounter in their natural environment, and measured both antigen-specific and total antibody concentration in the plasma of females, yolks, and nestlings. We traced the transfer of maternal antibodies from females to nestlings through the yolk and measured catabolisation of maternal antigen-specific antibodies in nestlings during early development. By utilizing measurements of non-specific and specific antibody levels in nestling plasma we were able to calculate the half-life of maternal antibodies in nestling plasma and the time point at which nestling were capable of synthesizing antibodies themselves. Based on the short half-life of maternal antibodies, the rapid production of endogenous antibodies by nestlings and the relatively low transfer of maternal antibodies to nestlings, our findings suggest that altricial-developing sparrows achieve immunologic independence much earlier than precocial birds.

**Conclusions/Significance:**

To our knowledge, this is the first in depth analyses performed on the adaptive immune system of a wild-passerine species. Our results suggest that maternal antibodies may not confer the immunologic protection or immune priming previously proposed in other passerine studies. Further research needs to be conducted on other altricial passerines to determine if the results of our study are a species-specific phenomenon or if they apply to all altricial-developing birds.

## Introduction

Immune-mediated maternal effects are believed to play an integral role in the disease resistance of mammalian [1]–[4] and avian offspring [5]–[9]. Maternal antibodies passively immunize immunologically naïve young against virulent antigens and parasites that the offspring might encounter in its immediate developmental environment [3], [4], [7], [10], [11]. Passerine birds are known to be ecologically, agriculturally, and environmentally relevant fixtures on six of the seven world continents and yet the effects of maternal antibodies on offspring development are not well defined for these altricial-developing species [12]. Studies that have examined humoral-immunologic development in passerines often designed experiments based on information gleaned from the primary literature using the domestic chicken (*Gallus gallus domesticus*) as a model of humoral immunity. Although this model has generated a great breadth of knowledge on the physiology and function of the avian immune system, its applicability to passerine birds is dubious, as passerines express an altricial mode of development that differs dramatically from the precocial mode of development. Altricial birds hatch from the egg with immature physiological functions. For example, thermoregulation, motor control, endocrine function, and neural control are not fully developed at the time of hatch [12]–[14]. Therefore, one might also predict major differences in both immunologic development and the role that maternal antibodies play in conveying protection from antigens early in life.

In precocial chickens, maternal antibodies are transferred across the follicular epithelium into the yolk during oogenesis [12], [15], [16]. There are three classes of avian immunoglobulins (IgY, IgM and IgA). Of these, IgY is transferred at the highest concentration and is functionally homologous to mammalian IgG [17]. IgA and IgM are found predominantly in the egg white of chicken eggs, but have been detected in the yolk at low concentration [18], [19]. Prior to hatch, maternal IgY is absorbed into embryonic circulation [16], where it confers passive immunity to immunologically immature hatchlings [4], [6], [7], [10], [11], [17], [20]. IgM is also absorbed into circulation, though at low concentrations (<1%) [17]. However, at present, there is no evidence supporting the uptake of IgA into nestling circulation from the yolk [18].

Both altricial and precocial species hatch with incompletely developed immune systems, making them vulnerable to pathogens in their environment [12], [21]–[24]. In precocial chickens, maternal antibodies persist in the chick's circulation for 14–21 days after hatch and have a half-life of 3–7 days [6], [25]–[27]. Chicks begin to produce their own endogenous antibodies 3–4 days after hatch and are not considered immunologically independent until all of the maternal antibodies have been catabolized from their system [12], [17], [27]–[30]. To our knowledge, the ontogeny and timing of immunologic independence in a passerine species remains unknown. This information is vital to the growing field of ecological immunology, which often focuses on passerine bird systems [8], [31]–[33].

In this study we describe a developmental profile of humoral immunologic independence in the altricial house sparrow by measuring total and antigen specific maternal antibodies in the yolk and nestling plasma as well as endogenous antibody synthesis by nestlings prior to fledge. We report the half-life of maternal antibodies, the time point at which nestlings synthesize their own antibodies and the time at which they become immunologically independent. Collectively, these results suggest that maternal antibodies may play a very limited role in immunologic development. Furthermore, we suggest that house sparrows may achieve immunologic independence at a much earlier stage of development than do precocial species such as the chicken.

## Results

### Female Immunoglobulin Production in Response to Vaccination

**Figure 1:**
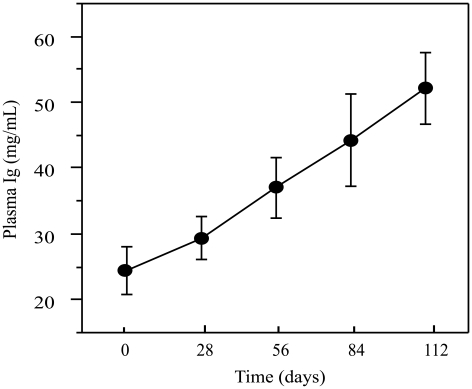
Female immunoglobulin production in response to vaccination with modified complete (mCFA) and incomplete (IFA) Freund's adjuvant. Mean ±SE plasma antibody concentrations (measured as mg/mL) in females vaccinated against DNP-KLH in mCFA (N = 20) or PBS (N = 15). Two booster shots containing DNP-KLH in IFA or PBS were given at 28-day intervals following the primary injection. A total of five bleeds were taken, 28-days apart, to measure total antibody concentration. Samples taken on day 0 reflect pre-vaccination total antibody titers. Samples were combined from both DNP-KLH and control treated females, as we found no effect of treatment on antibody concentration (P = 0.4586).

Vaccination with dinitrophenol keyhole limpet hemocyanin (DNP-KLH) versus phosphate buffered saline (PBS) did not have a significant effect on circulating antibody levels in female plasma (ANOVA: F_5, 111_ = 4.7602, P = 0.4586, R^2^ = 0.1834) and was removed as a model effect. Female plasma antibody levels increased more than two-fold over the course of the sample period after injection with modified complete Freund's adjuvant (mCFA) (ANOVA, F_4, 111_ = 5.8363, P = 0.0003, R^2^ = 0.1791; Figure 1: DNP-KHL-vaccination and control-injection combined). Female antibody concentrations were log-transformed to meet the assumption of normality. Pre-vaccination total Ig antibody titers ranged between 3.323 and 56.626 mg/mL with a mean of 23.096±13.365 mg/mL and a median of 19.445 mg/mL.

### Female KLH-Specific Antibody Production in Response to Vaccination

**Figure 2:**
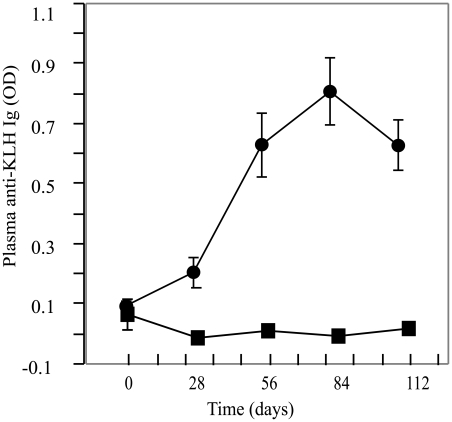
Female DNP-KLH-specific antibody response of adult females vaccinated against DNP-KLH in modified (mCFA) and incomplete (IFA) Freund's adjuvant. Mean ± SE of DNP-KLH-specific antibody levels (measured as optic density (OD)) in females vaccinated against DNP-KLH in mCFA (N = 20) or PBS (N = 15) on day 0. Two booster shots containing DNP-KLH in IFA or PBS were given at 28-day intervals following the primary injection. A total of five bleeds were taken, 28-days apart, to measure DNP-KLH-specific antibody levels. Samples taken on day 0 reflect pre-vaccination DNP-KLH specific antibody levels. Closed circles (•) represent DNP-KLH treated females and closed squares (▪) represent PBS treated females.

In contrast to total immunoglobulin levels, plasma levels of antibodies specific to dinitrophenol keyhole limpet hemocyanin (DNP-KLH) increased over time in vaccinated females versus controls (ANOVA: F_9, 100_ = 10.1280, P = 0.0001, R^2^ = 0.4768; Figure 2). We observed DNP-KLH-specific antibody levels increase seven-fold, with a peak between 2 and 3 months after vaccination. This confirms an antigen specific antibody response in females vaccinated against DNP-KLH.

### Transfer of Maternal Antibodies to Nestlings

There was a positive relationship between DNP-KLH-specific antibodies in the female plasma relative to the yolk (F_1,5_ = 12.6544, P = 0.0163, R^2^ = 0.7168; Figure S2, a) and plasma samples from hatch day 0 nestlings (F_1,8_ = 7.9484, P = 0.00225, R^2^ = 0.4984; Figure S2, b), which confirms the transfer of maternally derived antibodies to yolk and nestlings. There was no significant relationship between maternal total antibody levels and those in the yolk (F_1, 7_ = 0.1044, P = 0.7597, R^2^ = 0.0205; Figure S3, a), or nestling plasma on hatch day 0 (F_1, 7_ = 0.2396, P = 0.6395, R^2^ = 0.0331; Figure S3, b). The transfer of total immunoglobulin from female plasma to nestling plasma on hatch-day 0 was estimated to be 11.6±1.7% (N = 10; Table 1).

**Table 1:** Total antibody levels in the mother's plasma, yolk and nestling plasma.

**Female**	
**Plasma Ig (mg/mL)^1,2^**	24.4545±5.4043
**Yolk (mg/mL)^1,3^**	5.5884±1.8404
**Yolk (mg)^3,4^**	6.6233±2.3279
**% Transfer^5^**	11.5785±1.7430
**Nestling**	
**Plasma (mg/mL)^1^**	
**Day 0**	3.8282±1.2195
**Day 3**	1.0460± 1.2595
**Day 6**	2.3649±1.4082
**Day 9**	5.5843± 1.5426
**Day 12**	5.2140± 1.8438
**Day 15**	12.6370±1.6260

1 For mother and nestling plasma, antibody levels are presented as means ± SE.

2 Pre-vaccination antibody concentration in mother's plasma.

3 Yolk concentrations derived from whole yolks that underwent chloroform extraction.

4 Antibody weight/yolk as calculated from antibody concentration * egg mass.

5 Percent transfer of maternal antibody concentration from the mother to nestling.

### Nestling Antibody Profile

#### Total antibodies

**Figure 3:**
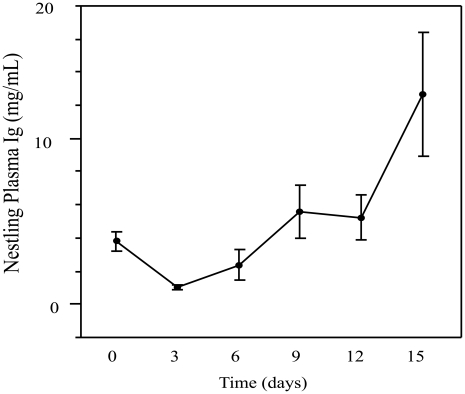
Total antibody concentration in nestlings at 0, 3, 6, 9, 12, and 15 days of age. Mean ± SE plasma antibody concentration (measured as mg/ml) from nestlings sampled at 0, 3, 6, 9, 12, and 15 days of age. The antibodies in hatch day 0 nestlings were assumed to be maternal in origin, while those from nestlings 3–9 day old are assumed as being a mixture of endogenous and maternal antibodies. Antibody concentrations measured in 12 and 15 day old nestlings were determined to be endogenous in origin.

Female treatment did not have a significant effect on nestling antibody levels over time (t (68) = -1.34, P = 0.1855) and was removed as a model effect from further analysis. Age had a significant effect on total immunoglobulin levels in nestling plasma (ANOVA: F_5, 63_ = 7.1616, P<0.0001, R^2^ = 0.3623; Figure 3). A comparison of means from each sampling time point revealed the lowest antibody titers occurred 3 days after hatch (1.0460±1.2595; Table S2). Antibody titers decreased between Hatch day 0 and day 3 and increased, relative to day 3, beginning on day 6 and continuing through day 15 (Figure 3, Table S2). Antibody titers on day 15 were significantly greater than those recorded at the five other time points (Table S2).

#### Maternal DNP-KLH-specific antibodies

**Figure 4:**
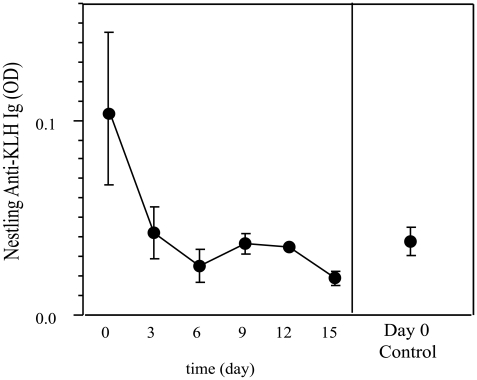
Maternal DNP-KLH-specific antibodies in nestlings sampled at 0, 3, 6, 9, 12, and 15 days of age. Mean ± SE of maternal DNP-KLH-specific antibodies (measured as optic density (OD)) in nestling plasma at 0, 3, 6, 9, 12, and 15 days of age. DNP-KLH specific antibodies are purely maternal in origin, as nestlings were not exposed to this antigen. The OD recorded for hatch day 0 nestlings was significantly different from control values obtained from hatch day 0 nestlings hatched from control females at P<0.05. All other samples taken from 3–15 day old nestlings did not significantly differ from the control at P<0.05.

The detection of DNP-KLH specific antibodies in nestling plasma (as estimated by optic density (OD)) was highest on hatch day 0 (0.1034±0.0192). This value was significantly greater than that in plasma from nestlings on day 0 that hatched from control females lacking DNP-KLH-specific antibodies (0.0386±0.01622; Table S3). However, DNP-KLH-specific antibody levels in nestling plasma from day 3 through day 15 were not significantly different from that in controls (Table S3). This indicates that DNP-KLH-specific maternally derived antibodies were undetectable by 3 days post-hatch and appeared to decrease rapidly with age (Figure 4). We did not detect a significant relationship of maternal DNP-KLH antibodies in nestling plasma over time (ANOVA: F_6, 40_ = 1.6945, P = 0.1475, R^2^ = 0.202664).

### Half-Life of Maternal Antibodies

The biological half-life of maternal antibodies in nestling plasma was calculated as 2.2±0.25 days (N = 17) with values ranging between 0.17 and 4.7 days. Defining 0.172 mg/mL as the minimum concentration of detectable maternal antibodies, post-hatch, maternal antibodies appear to persist in nestling plasma for up to 8.6±0.5 days post-hatch.

### Immunologic Independence

We defined immunologic independence as the time at which nestlings are capable of synthesizing antibodies and all maternal antibodies have been cleared from their plasma. In accordance with this definition, immunologic independence was estimated to occur between 8–10 days after hatch. This value is in accordance with the calculated half-life of 2.2±0.25 days maternal antibodies and the detection of endogenously produced antibodies in nestling circulation beginning 3–6 days after hatch.

## Discussion

In this study we report that maternal antibodies persist for a relatively short period of time during early development in an altricial-developing wild passerine bird. Previous research on the role of immune-mediated maternal effects in wild passerines has assumed that the ontogeny of immunocompetence in altricial passerines follows the time course described for precocial-developing *Gallus* species. Our results suggest that the ontogeny of the adaptive immune system in altricial-developing birds deviates from that of the chicken model.

### Half-Life of Maternal Antibodies

In newly hatched chickens, maternal antibodies have a half-life of approximately 3 days [27]. For antigen specific antibodies, a longer half-life has been reported (5–7 days) [6], [26], [34]. In our study with altricial house sparrows, maternal antibodies in the plasma of nestlings have a biological half-life of 2.2±0.25 days. This estimate is supported by the lack of detectable maternal DNP-KLH-specific antibodies in nestling plasma 3 days post-hatch (Figure 4). This half-life value is shorter than the 3 days reported for maternal West Nile Virus-specific antibodies in nestling house sparrows [31]. However, the assay used in that study measured antibody concentration qualitatively (% neutralizing activity) and samples were taken 1–7 days after hatch [31]. We have shown that house sparrow nestlings are capable of *de novo* synthesis of antibodies 3–6 days post-hatch, which, in regard to the study with West Nile Virus, suggests that the methods did not differentiate between maternal and endogenous antibodies when calculating the half-life. The sampling time of nestling plasma plays a critical role in determining the half-life of maternal antibodies. For example, using plasma samples obtained from hatch day 0 and day 6 nestlings, the estimated half-life is 9.045±0.65 days. This half-life is 4-times greater than the value we report (2.2±0.25 days), which is based on the dynamics of antibody levels across the period of nestling development. Thus, the frequency and timing of plasma sampling can have a significant effect on the perceived changes in antibodies derived from the mother versus produced by the chick.

We estimated maternal antibodies to persist in nestling plasma up to 8–9 days post-hatch. In chickens, maternal antibodies have been reported to remain detectable for 14–21 days after hatch [12], [17], [35]. This difference, in conjunction with the shorter half-life we report, strongly indicates that maternal antibodies persist in nestlings of the altricial house sparrow for much less time than in the precocial chicken. The absence of maternal DNP-KLH-specific antibodies 3 days after hatch suggest that maternal antibodies are limited in their ability to confer passive immunity against specific antigens in nestlings. This brings into question the suggestion in the literature that maternal antibodies play a central role in conferring immunologic protection to developing altricial nestlings [11], [31], [36]–[40]. Our data suggest that antigen-specific maternal antibodies are absent a few days prior to nestlings producing their own antibodies.

The fraction of maternal antibodies transferred to nestling plasma on hatch day 0 was estimated as 11.6±1.7%, which is about a 1/3 of what has been reported in chickens [17]. All females vaccinated against DNP-KLH tested positive for plasma DNP-KLH-specific antibodies and 85.7% of their eggs were positive for DNP-KLH-specific antibodies. However, only 44.4% of the chicks that hatched from DNP-KLH positive females tested positive for DNP-KLH-specific antibodies in their plasma on hatch day 0. This suggests that the transmission of antigen-specific antibodies from mother to offspring is limited or incomplete. Given that only 11.6% of the mothers total antibody concentration can be detected in newly hatched chicks the concentration of antigen-specific antibodies can be assumed to be even lower. The rapid decline of DNP-KLH specific antibodies in nestling serum is not surprising, given the short half-life paired with incomplete transfer of antigen-specific maternal antibodies. These results also bring into question the conclusions drawn from studies examining the effects that maternal antibodies have on nestling immunocompetence. If antigen specific antibodies are not transferred to the nestling or are catabolized before nestlings are sampled there is no definitive link between maternal antibodies and immune defense of the chicks [8], [32], [37], [38], [41], [42].

### Nestling Antibody Production

In this study we demonstrate that nestlings begin to synthesize antibodies between 3 and 6 days after hatch. This is consistent with what has been reported for chickens, where *de novo* synthesis begins 3–4 days post-hatch [30], [43]–[45]. Nestlings in our study showed a steep rise in plasma antibody levels, relative to day 3 titers, beginning on day 6 and continuing through the sampling period. By 15 days post-hatch nestling plasma antibody concentrations were well within the range observed for pre-vaccinated adult females (Range: 3.3231to 56.6262 mg/mL), suggesting that nestlings may be immunocompetent well before they fledge from the nest.

### Nestling Immunologic Independence

We defined immunologic independence as the time at which maternal antibodies were no longer detectable in nestling plasma and when *de novo* synthesis of antibodies began. Thus, in house sparrows, immunologic independence occurred at approximately 8–10 days of age. Based on these criteria, altricial-developing house sparrows may achieve immunologic independence much earlier than precocial chickens. Furthermore, given the rapid decline of DNP-KLH specific maternal antibodies over the first 3 days post-hatch, it is not unreasonable to question if maternal antibodies protect chicks at an early age. Studies on both altricial [31], [46] and precocial [35], [47], [48] avian species have reported that maternal antibodies may interfere with the offspring's ability to detect foreign antigen and, thus initiate an antigen-specific antibody response. It may be advantageous for both precocial and altricial species such as the house sparrow to achieve immunologic independence as early as possible given the high density of virulent antigens and parasites in their natural environment. Factors contributing to inter-specific variation in the timing of immunologic independence remain, as yet, undetermined.

### Conclusions

The results of this study suggest that altricial-developing passerines deviate from the model of avian immunity that is based on the precocial chicken. This has major implications for research examining immune-mediated maternal effects in offspring of altricial birds. A more in depth analysis of the ontogeny of the immune system needs to be undertaken before proper experiments can be designed and executed on wild passerines. Although maternal antibodies may protect nestlings during the period between hatch and endogenous antibody production, we have shown that there is a limited and incomplete transfer of novel antigen-specific maternal antibodies to nestlings, even when the mother is rigorously immunologically challenged. If, indeed, maternal antibodies have a limited protective effect for nestlings and do not confer much passive immunity, existing hypotheses need to be reexamined. Researchers might examine other pathways through which the mother may influence her offspring's immune defenses. It may be that genetic quality [9], [17], [49] and investment in parental care [41], [50], [51] play a more integral role in nestling immunologic development than immune-mediated maternal effects. Indeed, in chickens, it has been observed that some lines are more immunologically active than others and that these line differences are heritable [9], [17].

Our results indicate that maternal antibodies may not confer the immunologic protection or immune priming previously reported in other studies of passerine birds. Further research on other passerine species is warranted to determine if the results of our study on house sparrows are revealing a species-specific pattern or if they are applicable to other altricial-developing passerine and non-passerine birds. It may also be of interest to examine both proximate and ultimate level questions regarding deviations observed in the immunologic development of altricial passerines in relation to metabolism and growth rate, species-specific immunoglobulin structure, rearing environment, domestication, disease prevalence, and trade-offs between condition dependent life history traits.

## Methods

### Ethics Statement

All animal capture, handling and experimental procedures described herein were approved by the Institutional Animal Care and Use Committee (protocol #2551) at Washington State University. All collections were approved by US Fish and Wildlife Service Permit #04-202.

### Animal Capture and Care

During December of 2005 and January of 2006, 35 breeding pairs of wild house sparrows were captured with mist nets from agricultural sites near Pullman, WA (46°44′N 117°10′W). The birds were housed in five separate outdoor aviaries (1×2×2.5 m) with seven breeding pairs per aviary. Each aviary was equipped with 10 nest boxes, perches, nesting material (grass hay and pillow stuffing), and *ad libitum* access to food and water. Individuals were banded with a numbered aluminum ring and two colored bands for individual identification. The birds were allowed to acclimate to their surroundings for 30 days before experimentation began.

### Female Vaccination

Beginning in February, prior to egg laying, female house sparrows were placed on a vaccine schedule to stimulate antibody production. 20 females were injected subcutaneously with DNP-KLH in mCFA at a dose of 1 mg/kg of body mass. DNP-KLH is a novel, non-pathogenic antigen that females would not normally encounter in their environment. The detection of antibodies against DNP-KLH in the egg yolk and nestling plasma can, therefore, be assumed to be of maternal in origin. Modified Freund's adjuvant contains inactivated *Mycobacterium butyricum*, which acts as an immunostimulant for cell-mediated immunity. Individuals injected with this were expected to express increases in overall antibody titers regardless of antigen treatment. Two booster shots containing DNP-KLH in incomplete Freund's adjuvant (IFA), which lacks *M butyricum*, were injected at 28-day intervals. The remainder of the females (n = 15) received comparable injections of PBS for both the primary and booster shots. Experimental and control females were distributed across the five aviaries. To monitor total and DNP-KLH-specific antibody concentrations females were bled a total of five times at 28-day intervals beginning directly before they received their primary injection. We collected pre- and post-vaccination blood samples by puncturing the brachial vein with a 28-gauge needle and collecting the blood into heparinized microcapillary tubes (∼100 µL/bleed/individual). Blood samples were stored on ice for no more than two hours, whereupon plasma was separated from red blood cells by centrifuging samples at 9,000 rpm for 10 min and stored at −20°C until further analyses.

### Egg Sampling

Nest boxes were monitored daily for eggs. Once a female initiated a clutch the nest box was videotaped to determine parental identity. The day that an egg was laid it was marked with a non-toxic marker to determine laying order. The second laid egg from each clutch was collected and stored at −20°C until further processing for antibody concentrations in yolk.

### Antibody Extraction from Yolk

In preparation for assay, antibodies were extracted from the yolk using a modified chloroform-based method [17], [52]. Briefly, the yolk was separated from the albumen, washed with deionized water (dH_2_O) and weighed to the nearest 0.0001 g. A volume of PBS equal to the mass of yolk was added to the yolk sample and vortexed. To that suspension, a volume of chloroform equal to the volume of the PBS +Yolk solution was added, vortexed and then centrifuged at 1,000×g for 30 min at room temperature. After centrifugation, three distinct layers were observable: a lecithin layer on the bottom, a layer of emulsified yolk and chloroform in the middle, and a watery protein layer on the top, which contains antibodies. The clear, aqueous protein layer was removed and stored at −20°C until further analysis.

### Nestling Sampling

In previous aviary studies with house sparrows we observed a high incidence of nestling mortality during the first breeding season after their capture. This was subsequently attributed to impaired parental care and infanticide by neighboring pairs. To ameliorate this effect we fostered out aviary offspring to wild house sparrows breeding at a local field site at the University of Idaho's Sheep Center in Moscow, ID (46°43′N 116°59′W). Nest boxes had previously been mounted in three different barns in clusters of 20 and had been placed ∼1 m apart from each another. To the entrance of each box we attached a mesh-wire cylinder that allowed sparrows to freely enter and exit, but limited the ability of predators to gain access to the box. House sparrows were observed to occupy this site at a high density and readily occupied the boxes. Nest box activity was monitored daily at this site and on the day an egg was laid by a wild sparrow it was removed and replaced with a wooden dummy egg. Prior to this study we observed that house sparrows incubate dummy eggs for upwards of 3 weeks, thus giving us ample time to foster out aviary nestlings to wild breeding pairs. To insure nestling survival, eggs were removed from the aviary nest boxes 1–2 days before their estimated hatch date and placed in an incubator until hatch. Within 3 hours after their hatch they were transported to an active nest box at the field site. No more than 4 nestlings were placed in any one box and nestlings were paired according to age to reduce sibling competition.

Blood samples were collected every three days beginning at hatch (day 0) and ending on day 15, for a total of 6 bleeds. Hatch day zero and day three nestlings were bled from the jugular vein, while day 6–15 blood samples were obtained from the brachial wing vein. No more than 20 µL of blood was taken at any one time.

### Antibody Extraction and Purification

In preparation of producing secondary-antibodies specific to house sparrow-Immunoglobulins (HOSP-Ig) we extracted and purified antibodies from 30 house sparrow eggs following the protocol described by Hansen et al [53]. The recovered fraction of HOSP-Ig was separated from other water-soluble proteins in the yolk via thiophilic interaction chromatography as described in the manufacturers protocol (Clontech Laboratories, USA, #635616.). Per 3 mL column, 10 mL of extracted antibodies in sample buffer (50 mM sodium phosphate; 0.55 M sodium sulfate, pH 7.0) was loaded onto an equilibrated column (pH 7). The non-absorbed proteins were washed out using the equilibration buffer (50 mM sodium phosphate; 0.5 M sodium sulfate, pH 7.0) until the absorbance decreased to ∼0.030 AU, whereupon the bound antibodies were collected by running elution buffer (20 mM sodium phosphate, 20% glycerol, pH 7.0) over the column. The collected volume was loaded into a dialyses cassette to remove residual salts and glycerol from the solution. Finally, the purified suspension was lyophilized and stored at −20°C until further use. The presence of HOSP-Ig was confirmed using sodium dodecyl sulfate polyacrylamide gel electrophoresis (SDS-PAGE) on 12% slab-gels and stained with Coomassie Blue R-250. All of the eggs used for immunoglobulin extraction were obtained from a separate population of house sparrows who were not subject to experimentation.

### Secondary-Antibody Production

Polyclonal HOSP-Ig specific antibodies were generated in laboratory rats and rabbits for use in DNP-KLH specific and total antibody enzyme-linked immunosorbent assays (ELISA). The antiserum was generated against intact immunoglobulin molecules.

#### Rats

Three rats were vaccinated against purified HOSP-Ig. The lyophilized HOSP-Ig was redisolved in PBS (1 µg/µl) and emulsified with an equal volume of mCFA. The rats received one primary, subcutaneous injection of purified HOSP-Ig in mCFA at 50 µg of protein/100 µl emulsion and two booster shots containing HOSP-Ig in IFA at 4-week intervals. Rats were exsanguinated 4-weeks after the final booster shot.

Cross-reactivity between house sparrow antibodies and the rat antiserum was confirmed using western-blot analysis as described in Huber et al [54]. Briefly, purified antibodies were separated using SDS-PAGE and transferred on to a nitrocellulose membrane for immunoblotting. Chicken plasma diluted 1∶10 in ddH2O was included on the gel to act as negative control. Filters were blocked with casein blocking buffer for 1 h at room temperature and then washed three times in double deionized water (ddH2O). The blots were incubated for 1 hr at room temperature with rat-anti-house-sparrow-Ig (Rar**α**HOSP-Ig) diluted to 1∶1,000 in fluorescent treponemal antibody (FTA)-hemaaglutination buffer (pH 7.2) and then washed three times again with ddH2O. The blots were then incubated for another hour at room temperature with goat-anti-mouse plasma conjugated to horseradish peroxidase (G**α**M-hrp) (Bethyl Laboratories, Inc., Mongomery, TX) and washed a final three times with ddH2O. The blots were analyzed using enhanced chemiluminesence (Figure S1).

#### Rabbits

Sigma-Genosys was commissioned to build antiplasma specific to purified HOSP-Ig in rabbits. We supplied the company with 3.21 mg of purified lyophilized HOSP-Ig protein. Two rabbits received one primary injection of HOSP-Ig in mCFA followed by five booster shots containing HOSP-Igs in IFA at 14-day intervals. Cross reactivity between purified HOSP-Ig and the rabbit antiplasma was confirmed via indirect ELISA by Sigma-Genosys.

### DNP-KLH Specific Antibody ELISA

To detect DNP-KLH specific antibodies in female and nestling plasma as well as in the yolk we developed a DNP-KLH specific ELISA protocol. Prior to running the samples, the ELISA was optimized to determine the ideal concentration at which to dilute the capture-antigen, samples, secondary antibody, and detection antibody. The assay was validated via serially diluting pooled samples to show gradations in absorbance.

Immulon-4, 96-well plates were coated with 100 µL/well of DNP-KLH (0.1 mg/mL) in sodium bicarbonate coating buffer (0.05 M, pH 9.6) and incubated overnight in the cold room (4°C). Plates were washed three times with wash solution 200 µL/well (150 mM NaCl, 0.01% Tween-20). Each plate contained, in duplicate, a blank, a NSB (measured binding between the capture antigen and the secondary and detection antibodies), and DNP-KLH-positive and DNP-KLH-negative controls that were developed via pooled samples of plasma obtained from DNP-KLH vaccinated and unvaccinated female plasma, respectively. Positive and negative controls were serially diluted in sample buffer (FTA-hemmaglutination (pH 7.2), 10% casein) at concentrations of 1∶500, 1∶1000, and 1∶2000 and were run in duplicate. Female plasma samples, nestling plasma samples and the chloroform extracted yolk fraction were diluted 1∶1,000 in sample buffer and added, in duplicate, at a volume of 100 µL/well. Plates were incubated for 1 hr at 37°C on a rocker before being washed three times with wash solution. 100 µL of Rat**α**HOSP-Ig diluted at 1∶1000 in sample buffer was added to each well and the plates were again incubated for 1 hr at 37°C on a rocker before being washed three times. 100 µL/well of detection antibody, goat-anti-rat-horse-radish-peroxidase (G**α**R-hrp) (Invitrogen # A10549, Carlsbad, CA), was added to each wells at a dilution of 1∶40,000 of antibody to sample buffer and incubated for 1 hr at 37°C on a rocker before being washed a final three times. Finally, 100 µL of peroxidase substrate (2,2′-azino-bis(3-ethylbenzthiazoline-6-sulphonic acid (ABTS: Sigma cat. A1888) and peroxide was added to each well and the plates were covered with tinfoil and allowed to develop for 1 hr at room temperature before being read on a spectrophotometer using a 405-nanometer wavelength filter to measure OD.

Plates were standardized to control for between plate variations in absorbance. Prior to standardization, the NSB OD was subtracted from the samples and control ODs for each plate. Per plate, the positive ratio was calculated by dividing the average absorbance of the positive control by the average absorbance of the highest positive control reading observed amongst all of the plates, collectively. Final absorbance readings were calculated by dividing the average sample absorbance by the positive ratio for that plate.

### Total Immunoglobulin ELISA

We developed a quantitative immunoglobulin ELISA to measure total Igs in the plasma and yolk. The protocol was modified from a commercial quantitative ELISA kit used to detect chicken IgY in plasma and yolk samples (Bethyl Laboratories, Montgomery, TX). To generate a standard curve we weighed out lyophilized HOSP-Ig and diluted it to1000 ng/mL in sample buffer. From this we made seven additional standards, which were serially diluted to range in value from 3.12–200 ng/ml. Antisera against HOSP-Ig were developed in both rabbits and rats, with the Rabbit**α**HOSP-Ig acting as the capture antibody and Rat**α**HOSP-Ig acting as the secondary antibody. Previous attempts to use only antibody from a single species for both the capture and secondary antibody showed reduced binding and poor repeatability. The dilutions reported for the capture antibody, secondary antibody, and detection antibody were all optimized to increase binding efficiency and decrease NSB prior to running the samples. Yolk and plasma samples were diluted based on predetermined working dilutions for adult plasma, yolk and nestlings of each sample age group nestling age (Table S1). Immunlon−4, 96 well plates were coated with Rabbit**α**HOSP-Ig diluted 1∶1000 in coating buffer and left to incubate overnight in the cold room (4°C). Each plate contained, in duplicate, a blank, a NSB (measured binding between the capture antibody and the secondary and detection antibody), a positive control, and 8 standards. All of the controls, standards and samples were added in duplicate at a volume of 100 µL/well. The plates were incubated for 1 hr at 37°C on a rocker before being washed three times with wash solution. After washing, 100 µL of Rat**α**HOSP-Ig diluted 1∶1000 in sample buffer was added and the plates were again incubated for 1 hr at 37°C on a rocker before being washed three additional times. 100 µL of the detection antibody, G**α**Rat-hrp, was added to each well at a dilution of 1∶40,000 and the plates were incubated one last time for 1 hr at 37°C on a rocker before being washed a final three times. To each well, including the blank, 100 µL of ABTS developing reagent was added and the plates were covered with tinfoil and left to develop for 1 hr. At the end of 1 hr the plates were read on a spectrophotometer using a 405-nanometer wavelength filter to measure OD. The OD values were converted to ng/mL using softmax pro 4.8 (Molecular Devices Corp., Sunnyvale, CA). Samples were multiplied by their dilution factor and are presented as mg/mL. Yolk samples are presented as both mg/mL and total mg/yolk.

### Biological Half-Life of Maternal Antibodies in Nestlings

The biological half-life of maternal antibodies was determined using the following equation:
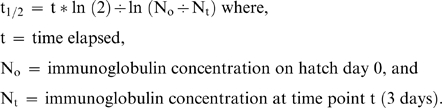



The DNP-KLH present in the yolk and nestling plasma was assumed to be maternal in origin, as nestlings were not exposed to the antigen naturally or experimentally. In nestling plasma the decline of maternal DNP-KLH was most pronounced between hatch day 0 and day 3. The concentration of total antibodies in nestling plasma decreased in parallel with maternal DNP-KLH specific antibodies and was, therefore, assumed to be maternal in origin. We, therefore, used the total immunoglobulin concentrations on day 0 and day 3 to calculate the biological half-life of maternal antibodies.

### Antibody Production in Nestlings

In nestling plasma, the total immunoglobulin concentration reached its nadir on day 3, post-hatch. Antibodies were determined to be endogenous in origin when an increase in antibody concentration was observed relative to the average concentration recorded on day 3. Any increase in total antibody concentration would have to come from the nestling.

### Immunologic Independence

Immunologic independence of nestlings was defined as the time point at which maternal antibodies were no longer detectable in the plasma in conjunction with the production of endogenous antibodies. This was determined by solving for *t* in the following formula:
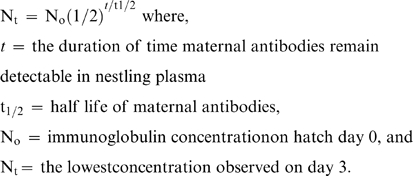



The lowest concentration of detectable antibodies on day 3 was selected as the value for N_t_ as it reflected lowest concentration of detectable maternal antibodies in nestling plasma.

### Statistical Analyses

All statistical analyses were carried out using JMP 7.0.1 (SAS Institute Inc., Cary, NC, U.S.A.). Pseudo-replication was avoided by analyzing data obtained from only one nestling per female, which was chosen at random. Data were analyzed using Student's t-tests for pairwise comparisons of female and nestling plasma immunoglobulin concentration, as measured at specific time points. Linear least-squares models were used to analyze the effects of time on specific (DNP-KLH) and total immunoglobulin concentrations in nestlings and breeding females. Correlations between female specific (DNP-KLH) and total immunoglobulin levels relative to those found in the yolk and nestling plasma on hatch-day 0 were also determined via linear least-squares analyses. The percent transfer of female total Ig to nestlings on hatch-day 0 was determined by dividing the concentration in nestling plasma on hatch-day 0 by the plasma concentration of total Ig in female plasma on the bleed date closest to the nestling's hatch date and multiplying that value by 100. Data are reported as the mean ± SE, and differences were considered statistically significant at P≤0.05. All data are expressed as the mean ± SE.

## Supporting Information

Figure S1 Western blot of purified house sparrow immunoglobulins and chicken plasma. Purified house sparrow immunoglobulins were obtained from thiophilic resin chromatography. Lyophilized immunoglobulin recoveries were diluted in sample buffer at 1 mg/mL. Both plasma and purified samples were loaded into columns at 1:10 and 1:20 dilutions. Images reflect cross reactivity between house sparrow immunoglobulins and the antiserum built in rats against purified Ig. Antiserum built in rats against house sparrow Ig did not cross react with chicken plasma.
(2.17 MB EPS)

Figure S2 Correlations between DNP-KLH-specific antibodies in mothers relative to a) the yolk (F1,8 = 7.9484, P = 0.00225, R2 = 0.4984) and b) nestling plasma on hatch day 0. (F1,8 = 7.9484, P = 0.00225, R2 = 0.4984). Antibody levels are expressed as the mean ± SE of optic density (OD).
(0.51 MB EPS)

Figure S3 Correlations between total immunoglobulin concentration in mothers relative to a) the yolk (F1, 7 = 0.1044, P = 0.7597, R2 = 0.0205) and b) nestling plasma on hatch day 0 (F1, 7 = 0.2396, P = 0.6395, R2 = 0.0331). Antibody concentration is expressed as the mean ± SE (mg/mL).
(0.59 MB EPS)

Table S1 Working dilutions used for plasma and yolk samples for the total antibody ELISA.
(0.04 MB DOC)

Table S2 P-values from Student's t-test comparing maternal DNP-KLH-specific antibodies in nestling plasma at 0, 3, 6, 9, 12, and 15 days of age.
(0.06 MB DOC)

Table S3 P-values from Student's t-test comparing plasma antibody concentrations in nestlings at 0, 3, 6, 9, 12, and 15 days of age.
(0.05 MB DOC)
