# Supplementary material for: Are Maternal Antibodies Really That Important? Patterns in the Immunologic Development of Altricial Passerine House Sparrows (Passer domesticus)
Source: PLoS One. 2010 Mar 11;5(3):e9639. doi: 10.1371/journal.pone.0009639 (PMC2836371; doi:10.1371/journal.pone.0009639)
Supplement: Table S1 — Working dilutions used for plasma and yolk samples for the total antibody ELISA. (0.04 MB DOC) [file pone.0009639.s004.doc]

| **Table S1. Working dilutions used for plasma and yolk** | | | |
| --- | --- | --- | --- |
| **samples for the total Antibody ELISA.** | | |  |
| **Sample** |  | **Working Dilution** | |
| **Maternal Plasma** | | 1:300,000 |  |
| **Nestling Plasma** |  |  |  |
| **Day 0** |  | 1:150,000 |  |
| **Day 3** |  | 1:25,000 |  |
| **Day 6** |  | 1:25,000 |  |
| **Day 9** |  | 1:25,000 |  |
| **Day 12** |  | 1:25,000 |  |
| **Day 15** |  | 1:25,000 |  |
| **Yolk^1^** |  | 1:25,000 |  |
|  |  |  |  |
| **^1^**Yolk dilutions refer to the recovered fraction | | |  |
| collected after yolk chloroform extractions were | | |  |
| performed. |  |  |  |
