# Supplementary material for: Are Maternal Antibodies Really That Important? Patterns in the Immunologic Development of Altricial Passerine House Sparrows (Passer domesticus)
Source: PLoS One. 2010 Mar 11;5(3):e9639. doi: 10.1371/journal.pone.0009639 (PMC2836371; doi:10.1371/journal.pone.0009639)
Supplement: Table S2 — P-values from Student's t-test comparing maternal DNP-KLH-specific antibodies in nestling plasma at 0, 3, 6, 9, 12, and 15 days of age. (0.06 MB DOC) [file pone.0009639.s005.doc]

| **Table S3. P-values from Student's t-test comparing maternal DNP-KLH-specific antibody levels in nestling plasma** | | | | | | |  |
| --- | --- | --- | --- | --- | --- | --- | --- |
| **at 0, 3, 6, 9, 12, and 15 days of age.^1^** | | |  |  |  |  |  |
| **Day** | **0** | **3** | **6** | **9** | **12** | **15** | **Control^3^** |
| **0** | -----^2^ | 0.0398 | 0.0177 |  |  |  | 0.0138 |
|  |  |  |  |  |  |  |  |
| **3** |  | ----- |  |  |  |  |  |
|  |  |  |  |  |  |  |  |
| **6** |  |  | ----- |  |  |  |  |
|  |  |  |  |  |  |  |  |
| **9** |  |  |  | ----- |  |  |  |
|  |  |  |  |  |  |  |  |
| **12** |  |  |  |  | ----- |  |  |
|  |  |  |  |  |  |  |  |
| 15 |  |  |  |  |  | ----- |  |
|  |  |  |  |  |  |  |  |
| **Control^3^** |  |  |  |  |  |  | ----- |
| **^1^**Pairwise comparisons were considered significane at P<0.05. Only p-values less than 0.05 are presented in the table. | | | | | | |  |
| ^2^Dashed lines represent time points that were not compared. | | | |  |  |  |  |
| **^3^**Control plasma obtained from nestling on hatch day 0 that hatched from control females. | | | | |  |  |  |
|  |  |  |  |  |  |  |  |
